# 4518 Metabolomic Identifiers Predictive of Adverse Events due to Acetaminophen Administration

**DOI:** 10.1017/cts.2020.337

**Published:** 2020-07-29

**Authors:** Brandon Joseph Sonn, Kennon Heard, Andrew Monte

**Affiliations:** 1 University of Colorado at Denver

## Abstract

OBJECTIVES/GOALS: Acetaminophen (Tylenol, APAP) toxicity has been well documented and well explored over the last 50 years. However, there has been no investigation into identification of specific metabolites that can *predict* which patients will have adverse reactions to therapeutic doses of APAP. METHODS/STUDY POPULATION: 205 subjects recruited from the Denver, CO community received the highest recommended daily dosing of APAP, 4 grams, for 16 days. Subjects were grouped by 1) alanine aminotransferase (ALT) at any monitored time point above 60units/L (n = 20) vs 2) no increase in ALT at any time point (n = 185). Blood was collected at days 0, 4, 7, 16, and 31. Samples were run on ultra-high performance liquid chromatography mass spectrometry with 27 heavy-labeled standards for metabolites documented to be associated with APAP metabolism. Data will be analyzed to look for significant changes in metabolite and demographic variable expressions using t-tests, chi square and logistic regression, as appropriate. RESULTS/ANTICIPATED RESULTS: It is expected that there will be greater elevations of conjugated non-toxic APAP metabolites (APAP-glucuronide, APAP-sulfate) in subjects whose ALT did not elevate because of successful hepatoprotection. Conjugated APAP metabolites are expected to only be present in samples taken after APAP therapy initiation confirming exposure as compared to being predictive of toxic response. Increases in lactate and cysteine in pre-exposure samples would allow for prediction of APAP toxicity as they are expected to have increased expression in subjects whose ALT became elevated which is indicative of increased hepatic damage due to oxidative damage. DISCUSSION/SIGNIFICANCE OF IMPACT: Identification of metabolites and/or demographic factors associated with toxic response to APAP prior to administration could advise APAP recommendations. Quantification of post-APAP administration metabolites would identify extent of successful hepatoprotective mechanisms.

